# Downregulated E-Cadherin Expression Indicates Worse Prognosis in Asian Patients with Colorectal Cancer: Evidence from Meta-Analysis

**DOI:** 10.1371/journal.pone.0070858

**Published:** 2013-07-29

**Authors:** Xin He, Zhigang Chen, Minyue Jia, Xiaoying Zhao

**Affiliations:** 1 Department of Hematology, the Second Affiliated Hospital, Zhejiang University School of Medicine, Hangzhou, China; 2 Department of Oncology, the Second Affiliated Hospital, Zhejiang University School of Medicine, Hangzhou, China; 3 Department of Endocrinology, Second Affiliated Hospital, Zhejiang University School of Medicine, Hangzhou, China; Health Canada and University of Ottawa, Canada

## Abstract

**Background:**

Epithelial-mesenchymal transition (EMT) plays a crucial role in the progression and aggressiveness of colorectal carcinoma. E-cadherin is the best-characterized molecular marker of EMT, but its prognostic significance for patients with CRC remains inconclusive.

**Methodology:**

Eligible studies were searched from the PubMed, Embase and Web of Science databases. Correlation between E-cadherin expression and clinicopathological features and prognosis was analyzed. Subgroup analysis was also performed according to study location, number of patients, quality score of studies and cut-off value.

**Principal Findings:**

A total of 27 studies comprising 4244 cases met the inclusion criteria. Meta-analysis suggested that downregulated E-cadherin expression had an unfavorable impact on overall survival (OS) of CRC (n = 2730 in 14 studies; HR = 2.27, 95%CI: 1.63–3.17; Z = 4.83; P = 0.000). Subgroup analysis indicated that low E-cadherin expression was significantly associated with worse OS in Asian patients (n = 1054 in 9 studies; HR = 2.86, 95%CI: 2.13–3.7, Z = 7.11; P = 0.000) but not in European patients (n = 1552 in 4 studies; HR = 1.14, 95%CI: 0.95–1.35, Z = 1.39; P = 0.165). In addition, reduced E-cadherin expression indicated an unfavorable OS only when the cut off value of low E-cadherin expression was >50% (n = 512 in 4 studies; HR = 2.08, 95%CI 1.45–2.94, Z = 4.05; P = 0.000). Downregulated E-cadherin expression was greatly related with differentiation grade, Dukes' stages, lymphnode status and metastasis. The pooled OR was 0.36(95%CI: 0.19–0.7, Z = 3.03, P = 0.002), 0.34(95%CI: 0.21–0.55, Z = 6.61, P = 0.000), 0.49(95%CI: 0.32–0.74, Z = 3.02, P = 0.002) and 0.45(95%CI: 0.22–0.91, Z = 3.43, P = 0.001), respectively.

**Conclusions:**

This study showed that low or absent E-cadherin expression detected by immunohistochemistry served as a valuable prognostic factor of CRC. However, downregulated E-cadherin expression seemed to be associated with worse prognosis in Asian CRC patients but not in European CRC patients. Additionally, this meta-analysis suggested that the negative threshold of E-cadherin should be >50% when we detected its expression in the immunohistochemistry stain.

## Introduction

Colorectal cancer (CRC) is the third most common cause of cancer-related deaths worldwide, and its 5-year survival rate ranges from 90% for stage1 patients to 10% for metastatic cases [1]. Thus, distant metastases formation is the decisive and the most lethal event during the disease course. In fact, about 25% of CRC patients present with Liver metastases at the time of diagnosis. Although Liver metastasis may be successfully treated by surgical resection, more than two thirds experience relapse [2]. Furthermore, 30–40% of cases will unfortunately develop metastases within 2 years after the resection of the primary tumour. Therefore, it is important to uncover the biological mechanisms underlying metastases of CRC and formulate strategies to intervene in this process.

Mounting evidence suggests that epithelial-mesenchymal transition (EMT) plays a crucial role in the progression and aggressiveness of colorectal carcinoma [3]–[6]. EMT was first recognized as an essential component of embryonic development, tissue remodeling, and wound repair [7]. Later, EMT was reported to participate in the progression and metastases of many epithelial tumors [8]. During the process of EMT, epithelial cells actively downregulate cell–cell adhesion systems, lose polarity, and acquire a mesenchymal phenotype. This phenotype enables tumor cells to infiltrate surrounding tissues, and thus license these cells to metastasize in distant sites. Several markers have been recognized as indicators of EMT, such as E-cadherin, vimentin, N-cadherin, and Snail [8], [9]. E-cadherin is the best-characterized molecular marker of EMT and loss of E-cadherin expression is an EMT hallmark [9], [10]. Therefore, E-cadherin is expected to be a useful biomarker associated with invasiveness, poor differentiation and malignant phenotype in CRC. However, the correlation between the expression of E-cadherin detected by immunohistochemistry and patient survival remains controversial, and the number of cases enrolled in numerous studies published was not large enough. Therefore, it is necessary to analyze the data of E-cadherin systematically in CRC to draw a reasonable conclusion about its prognostic significance.

In this study, we performed a meta-analysis to investigate E-cadherin expression and the prognosis of patients with CRC to determine whether low E-cadherin expression is associated with poor outcome and clinicopathologic characteristics of CRC.

## Methodology

### Literature search

We carried out a search of the PubMed, Embase and Web of Science databases using the terms: “E-cadherin”, “CDH1”, “colorectal neoplasms”, “colorectal Cancer”, “colon cancer” “rectal cancer”, “prognosis” with all possible combinations. The references of all the studies were manually searched for additional eligible studies. Review articles and bibliographies of other pertinent article were also inspected to find related articles.

### Inclusion and exclusion criteria

The inclusion criteria in the meta-analysis were as follows: (1) to evaluate E-cadherin expression by immunohistochemistry in the human CRC tissues; (2) to assess the relationships between E-cadherin expression and CRC pathological features or prognosis; (3) to be published in English language; (4) to provided sufficient information to estimate hazard ratio (HR) or odds ratio (OR) and their 95% confidence intervals (CIs).

The articles were not in the scope of our analysis if they met the following criteria: (1) letters, reviews, conference abstracts, case reports; (2) articles which don't offer enough data to calculate the HR about overall survival (OS); (3) articles published in non-English; (4) overlapping articles.

### Data extraction and assessment of study quality

Two investigators (HX and JMY) reviewed each eligible study and extracted following data: the first author's name, year of publication, country of origin, number of patients, gender of patients, tumor site, disease stage, antibody source, cut-off value, condition of adjuvant therapy and survival data. Controversial problems were arbitrated by the third investigator (ZXY). Newcastle–Ottawa quality assessment scale was used to assess the quality of each study [11].

### Statistical analysis

Odds ratios (ORs) and their 95%CIs were combined to evaluate the association between E-cadherin expression and clinicopathological factors, such as differentiation grade, Dukes' stages, depth of invasion, lymphnode status and metastasis. For the pooled analysis of E-cadherin expression on survival outcome, HRs and its 95% CI were the recommended summary statistics for meta-analysis of OS. If these statistical variables were described in a literature, we pooled it directly; otherwise, they were calculated from available numerical data in the articles according to the methods described by Parmar [12]. An observed OR<1 implies unfavorable parameters for the group with decreased E-cadherin expression. An observed HR>1 implies worse survival for the group with decreased E-cadherin expression. The impact of decreased E-cadherin expression on survival or clinicopathological factors was considered to be statistically significant if the 95%CI did not overlap with 1. Heterogeneity across studies was assessed by Chi- square based Q statistical test [13]. And the I^2^ statistic to quantify the proportion of the total variation, which is due to inter-study heterogeneity rather than sampling error and is measured from 0% to 100% [14]. A P>0.10 for the Q-test indicated a lack of heterogeneity among the studies, then the pooled ORs and HRs estimate of each study were calculated by the fixed-effects model (the Mantel-Haenszel method) [15]. Otherwise, the random-effects model (the DerSimonian and Laird method) was used [16]. Egger's test was used to examine the possibility of publication bias. Publication bias was indicated when p value of Egger's test <0.05. The statistical analyses were performed using STATA version 12.0 software (Stata Corporation, Collage Station, Texas, USA). All the P values were for a two-side test and considered statistically significant when p<0.05.

## Results

### Description of studies

**Figure 1:**
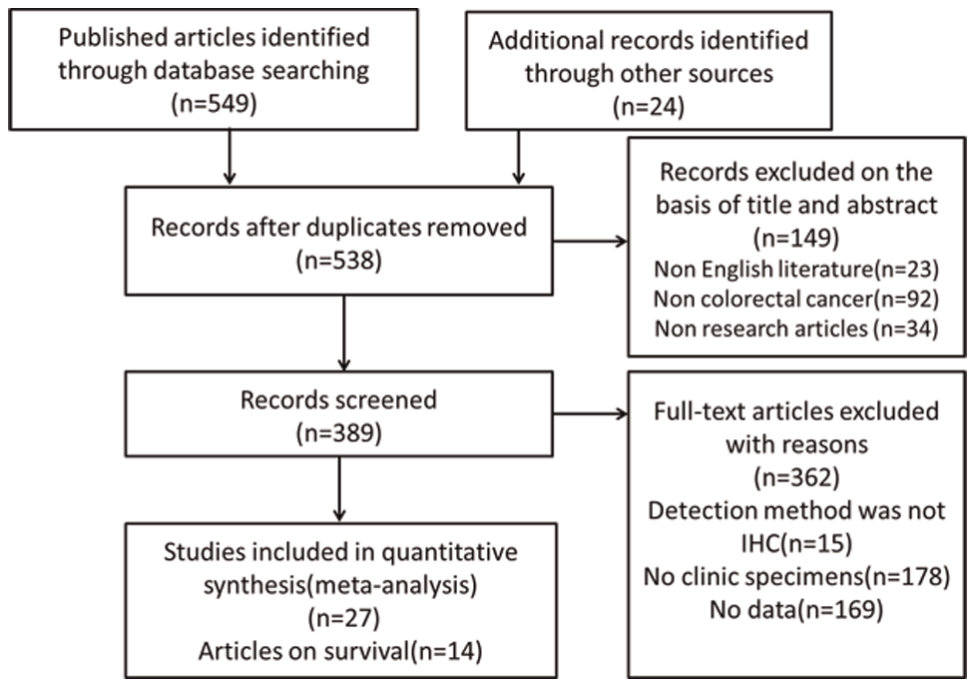
Flow diagram of studies selection procedure.

A total of 549 studies were identified from a search of the above databases using the search strategy as described above (**Figure** **1**). After scrutinizing the abstracts and full-text of these studies, a total of 27 eligible studies were ultimately chosen in this meta-analysis [17]–[43]. The clinical features of these 27 included studies were summarized in **Table** **1**. These studies were published from 1996 to 2012, and total 4244 CRC patients were enrolled and investigated the relationship between E-cadherin expression and pathological features or OS. Sample sizes ranged from 37 to 1164 patients. 11 studies enrolled less than 100 patients and 4 studies included more than 200 patients. Of these 27 studies, 7 studies were conducted in Japan, 4 each in China and Greece, 2 in Turkey, 1 each in Hungary, Korea, Italy, Roumania, European, Argentina, Russia, Norway, Sweden and England. 17 studies selected the percentage of negative staining as the cut-off point, including 10 studies more than 50%, 4 studies less than 50% and 3 studies 50%.

**Table 1:** Characteristics of studies included for the meta-analysis.

First author	Year	Country	Patient(M/F)	Antibody source	Definition of E-cadherin negative	HR of OS(95%CI)	Relationship with survival	Quality score
Lu	2012	China	136(84/52)	BD	Multiplying the intensitys core by expressions core ≤3	NA	NA	4
Andras	2012	Hungary	100(52/48)	Transduction	25%	1.1(0.55–2.198)	No	3
Chen	2012	China	60(NA)	Santa Cruz	50%	NA	NA	6
Lampropoulos	2012	Greece	195(103/92)	Santa Cruz	75%	NA	NA	6
Ozguven	2011	Turkey	60(38/22)	Neomarkers	50%	NA	NA	6
Kang	2011	Korea	301(168/133)	Transduction	Multiplying the intensitys core by expressions core ≤3	10.753(2.95–40)	Yes	4
Fang	2010	China	142(80/62)	Dako	30%	NA	NA	6
Karamitopoulou	2010	Greece	82(39/43)	Dako	60%	1.389(0.714–2.5)	No	3
Aresu	2010	Italy	44(22/22)	Transduction	50%	NA	NA	6
Filiz	2009	Turkey	138(83/55)	Labvision	Lightly/moderately staining or negtive staining	2.024(1.21–3.378)	Yes	6
Pap	2009	Roumania	149(87/62)	Labvision	Summing the intensitys core and expressions core = 0	NA	NA	6
Chen	2008	China	60(36/24)	Beijing Zhongshan Golden Bridge	Summing the intensitys core and expressions core ≤2	3.311(0.993–11.111)	No	6
Zlobec	2007	European	1164(NA)	Dako	5%	1.09(0.89–1.319)	No	2
Nqan	2007	Japan	140(79/61)	Vector	75%	2.849(1.362–5.952)	Yes	4
Shioiri	2006	Japan	138(83/55)	Takara	Weaker staining than normal or negtive staining	2.247(1.164–4.348)	Yes	6
Shiono	2006	Japan	86(NA)	NA	NA	4.237(1.289–13.889)	Yes	2
Roca	2006	Argentina	84(47/37)	BD	90%	2.38(1.316–4.348)	Yes	7
Bravou	2005	Greece	125(NA)	BD	70	NA	NA	7
Delektorskaya	2005	Russia	129(NA)	Novocastra	75%	NA	NA	3
Bondi	2006	Norway	206(96/110)	Zymed	70%	2.1(0.75–5.917)	No	5
Fernebro	2004	Sweden	269(173/96)	Dako	Weak or absent staining	NA	NA	5
Garinis	2003	Greece	37(20/17)	Santa Cruz	70%	NA	NA	6
Aoki	2003	Japan	82(44/38)	Transduction l	20%	3.597(1.075–12.048)	Yes	8
Ikeguchi	2000	Japan	105(58/47)	Takara	20%	3.448(1.135–10.526)	Yes	8
Nanashima	1999	Japan	44(29/15)	Takara	Absent staining	4(1.292–12.346)	Yes	7
Ilyas	1997	England	68(NA)	Dako	75%	NA	NA	5
Mohri	1996	Japan	100(59/41)	Sigma	90%	NA	NA	7

NA, not available; HR, hazard ratio; OS, overall survival; 95%CI, 95% confidence interval.

### Methodological quality of the studies

The qualities of 27 eligible studies included in our meta-analysis were assessed according to the Newcastle–Ottawascale (NOS). NOS assessed eight items of methodology, which were categorized into the three dimensions of selection, comparability, and outcome. For quality, scores ranged from 0 (lowest) to 9 (highest), and studies with scores of 6 or more were rated as high quality. 16 included studies obtained scores of 6 or more in methodological assessment, indicating that they were of high quality (**Table** **1**).

### Impact of E-cadherin expression on overall survival of colorectal cancer

**Figure 2:**
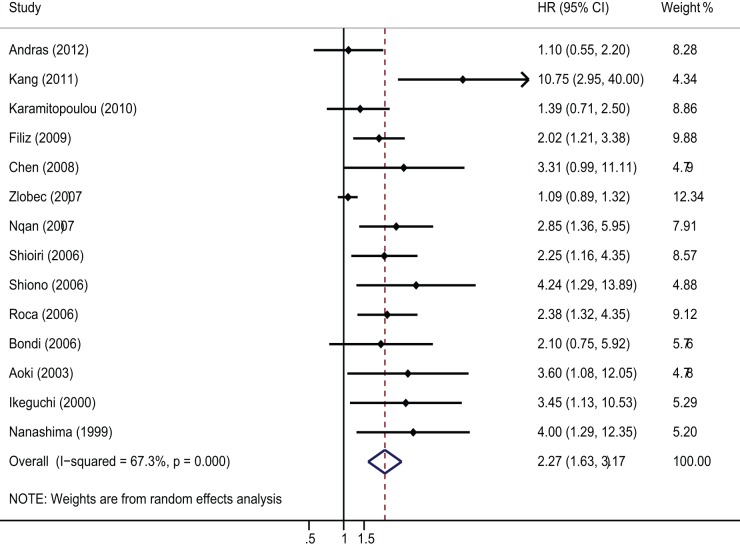
Forrest plot of Hazard ratio (HR) for the association of E-cadherin expression with overall survival (OS). HR>1 implied worse survival for the group with negative/decreased E-cadherin expression and loss of E-cadherin was significantly with the worse prognosis of CRC patients.

The meta-analysis was performed on 14 studies assessing the association of E-cadherin expression with OS. The pooled HR was 2.27, (95%CI: 1.63–3.17; Z = 4.83; P = 0.000) (**Figure** **2**) with heterogeneity (I^2^ 67.3% P = 0.000). It suggested that loss of E-cadherin was significantly with the worse prognosis of CRC and low or absent E-cadherin expression was a valuable prognostic factor in CRC. Moreover, we also performed subgroup analysis by study location, number of patients, quality score and cut-off value. The results showed that the significant relation between low E-cadherin expression and OS was exhibited especially in Asian countries (HR = 2.86 95%CI 2.13–3.7, Z = 7.11; P = 0.000). Additionally, reduced E-cadherin expression indicated an unfavorable OS only when the cut off value of low E-cadherin expression >50% (n = 512 in 4 studies; HR = 2.08, 95%CI 1.45–2.94, Z = 4.05; P = 0.000) (**Table** **2**). Subgroup analysis on other factors such as quality score, number of patients did not alter the significant prognostic impact of downregulated E-cadherin expression (**Table** **2**). In a sensitivity analysis, we removed one study at a time and evaluated the rest, the summary HR ranged from 2.07 (95% CI: 1.52 – 2.82) after excluding the study of Kang et al to 2.45 (95% CI: 1.71 – 3.5) after excluding the study of Andras et al (**Table** **3**).

**Table 2:** Stratified analysis of pooled hazard ratios of colorectal cancer patients with reduced E-cadherin expression.

					Heterogeneity	
Stratified analysis	Number of studies	Number of patients	Pooled HR(95%CI)	P value	I^2^(%)	P value
Study location						
Asia	9	1054	2.86(2.13–3.7)	0	0	0.507
Europe	4	1552	1.14(0.95–1.35)	0.165	0	0.587
Number of patients						
>100	8	2192	2.08(1.33–3.23)	0.001	73.7	0
<100	6	538	2.38(1.67–3.33)	0	5.5	0.381
Cut off value						
≤50%	4	1451	1.56(0.89–2.7)	0.116	59.7	0.059
>50%	4	512	2.08(1.45–2.94)	0	0	0.477
Quality score						
≤5	7	2079	1.27(1.2–3.33)	0.007	73.1	0.001
>5	7	651	2.5(1.85–3.33)	0	0	0.889

**Table 3:** HRs (95% CI) of sensitivity analysis for the meta-analysis.

Study omitted	Estimated HR	low value of 95%CI	High value of 95%CI
Andras (2012)	2.4472163	1.7096629	3.5029523
Kang (2011)	2.071373	1.520251	2.8222878
Karamitopoulou (2010)	2.4139702	1.6753392	3.4782522
Filiz (2009)	2.3332052	1.6147903	3.3712401
Chen (2008)	2.2308643	1.5846187	3.1406643
Zlobec (2007)	2.3679984	1.813562	3.0919354
Nqan (2007)	2.2296138	1.5749115	3.1564808
Shioiri (2006)	2.2920065	1.6029922	3.2771797
Shiono (2006)	2.1931612	1.5658236	3.0718379
Roca (2006)	2.276603	1.5928479	3.253871
Bondi (2006)	2.2962635	1.6179354	3.2589839
Aoki (2003)	2.2190771	1.5786371	3.119338
Ikeguchi (2000)	2.2186153	1.5769041	3.1214669
Nanashima (1999)	2.1959348	1.5661801	3.0789113
Combined	2.2725907	1.6285183	3.1713911

### Correlation of E-cadherin expression with clinicopathological parameters

**Figure 3:**
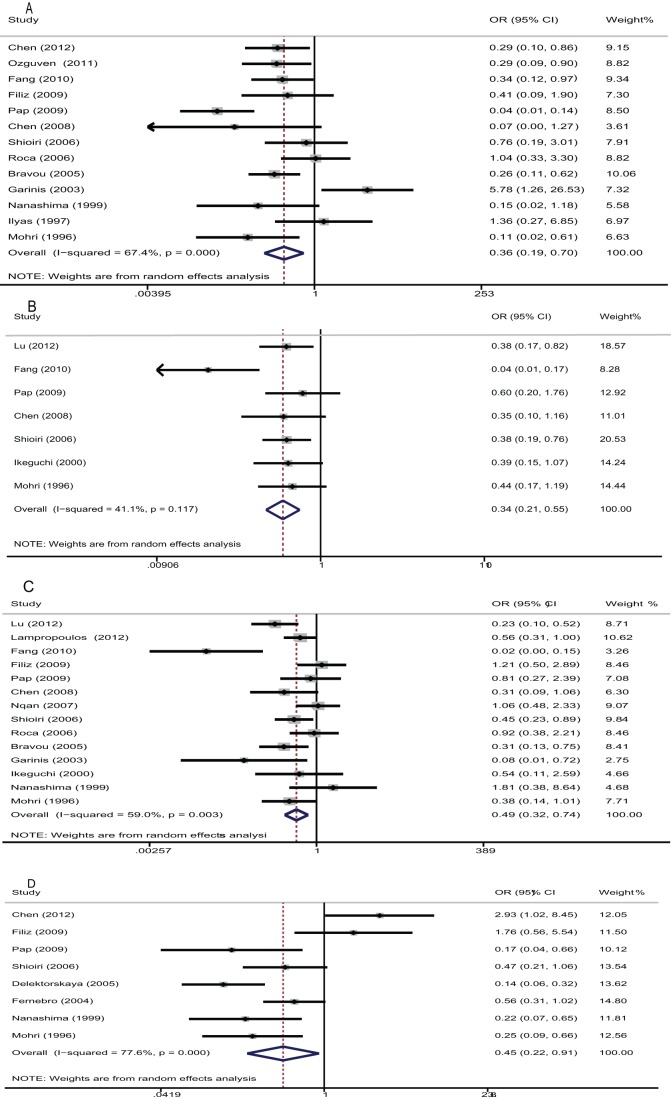
Forrest plot of odds ratios (ORs) for the association of E-cadherin with clinicopathological features. **A.** ORs with corresponding 95% CIs of the E-cadherin expression with differentiation grade. OR<1 suggested that unfavorable parameters for the group with negative/decreased E-cadherin expression and downregulated E-cadherin expression was associated with poor differentiation of CRC. **B.** ORs with corresponding 95% CIs of the E-cadherin expression with Dukes' stages. OR<1 indicated that low E-cadherin expression was associated with advanced Dukes' stage of CRC. **C.** ORs with corresponding 95% CIs of the E-cadherin expression with lymphnode metastasis. **D.** ORs with corresponding 95% CIs of the E-cadherin expression with distant metastasis.

Thirteen studies evaluated the correlation of E-cadherin expression with differentiation grade. The pooled OR was 0.36(95% CI: 0.19–0.7, Z = 3.03, P = 0.002) with heterogeneity (I^2^ 67.4% P = 0.000) (**Figure** **3A**) (**Table** **4**), and it suggested that downregulated E-cadherin expression was associated with differentiation of CRC. Seven studies assessed the correlation of E-cadherin expression with Dukes' stages. The pooled OR was 0.34(95%CI: 0.21–0.55, Z = 6.61, P = 0.000), indicating that low E-cadherin expression was associated with progression of CRC (**Figure** **3B**) (**Table** **4**). We also assessed the association between E-cadherin expression and lymphnode status and metastasis. The pooled OR was 0.49(95% CI: 0.32–0.74, Z = 3.35, P = 0.001) and 0.45(95% CI: 0.22–0.91, Z = 2.24, P = 0.025) (**Figure** **3C and 3D**) (**Table** **4**), which suggested that downregulated E-cadherin expression was associated with metastasis of CRC. Furthermore, there was no significant association between E-cadherin expression with AJCC stage and depth of invasion. The pooled OR was 0.74 (95% CI: 0.54–1.0, Z = 0.18, P = 0.051), and 0.47(95% CI: 0.19–1.16, Z = 1.64, P = 0.1), respectively (**Table** **4**).

**Table 4:** E-cadherin expression and clinicopathological features of colorectal cancer.

					Heterogeneity	
Clinicopathological features	Number of studies	Number of patients	Pooled OR (95%CI)	P value	I^2^(%)	P value
Differentiation grade (G1/G2 vs. G3/G4)	13	1205	0.36(0.19–0.7)	0.002	67.4	0
Dukes' stages (A/B vs. C/D)	7	725	0.34(0.21–0.55)	0	41.1	0.117
Lymphnode status (No vs. Yes)	13	1593	0.49(0.32–0.74)	0.001	59	0.003
Metastasis (No vs. Yes)	8	1027	0.45(0.22–0.91)	0.025	77.6	0.000
AJCC stage (I/II vs. III/IV)	7	831	0.74 (0.54–1.0)	0.051	4	0.396
Depth of invasion (No vs. Yes)	5	656	0.47(0.19–1.16)	0.1	49.3	0.096

### Publication bias

Egger's test indicated that there was no evidence of significant publication bias after assessing the funnel plot (**Figure S1**–**S5**) for the studies included in our meta-analysis.

## Discussion

E-cadherin is a well-described cosuppressor that was important in cell adhesion. Decreased production of E-cadherin, one of the central events underlying EMT, has been linked to increased invasiveness in several cancers [9], [44]–[46]. However, there is no consensus on the association between reduced E-cadherin expression detected by IHC and poor survival in patients with CRC at present. Meta-analysis is a systematical approach applied widely to the evaluation of prognostic indicators in different trials. Thus, we performed a quantitative meta-analysis to determine the association between E-cadherin expression and the survival and clinicopathological features of CRC.

To explore the connection with the CRC survival, our analysis combined the outcomes of 14 studies comprising 2730 CRC patients, indicating that the relationship between reduced E-cadherin expression and worse prognosis of CRC was obviously (HR = 2.27, 95%CI: 1.63–3.17; Z = 4.83; P = 0.000). In addition, the significant relationship was not changed in a sensitivity analysis removing each study. Subgroup analysis revealed that low E-cadherin expression was only significantly associated with poor prognosis in Asian countries, while not in European countries. Recently, E-cadherin was also reported to be related with gastric cancer or non-small cell lung cancer among Asians but not Europeans [47], [48]. These observations concurred with our finding and suggested that E-cadherin expression could be racial different as a prognostic factor. In addition, we found that the cut off value of low E-cadherin expression also altered the prognostic significance. The prognostic value of low E-cadherin expression existed when the threshold was >50% rather than ≤50%. Moreover, significant correlations were also observed between E-cadherin expression and clinicopathological features including differentiation grade, Dukes' stages, lymphnode status and metastasis.

In this meta-analysis, we had dealt with highly significant heterogeneity among the 27 studies. Although we used random effects models to analyze the data, heterogeneity was still a potential problem to affect meta-analysis results. Meanwhile, we only chose studies with methods of immunohistochemisty to reduce heterogeneity as soon as possible, but source and dilutions of primary antibodies, evaluation standards, clinicopathological parameters, study location, number of patients, sex and age of patients and quality score were quite different, which contributed to the heterogeneity inevitably. When the analysis on OS was performed without consideration of other factors, obvious heterogeneity was detected (I^2^ = 67.3%, P = 0.000). Thus, we performed stratified analysis according to study location, quality score, number of patients and cut-off value to identify the source of the great heterogeneity, and found that when the analysis was carried out on the basis of study location, heterogeneity disappeared. Therefore, the heterogeneity in this study might be explained by the patient ethnicity.

Meanwhile, there were some limitations in this meta-analysis. First, the study included in our meta-analysis was restricted only to articles published in English, which probably brought about additional bias. Second, the credibility of HRs calculated from data or extracted from survival curves might be less than that of direct analysis of variance.

In summary, we showed that low or absent E-cadherin expression was significantly connected with metastasis and worse prognosis of CRC in Asian patients in this study. Furthermore, a cut off value of more than 50 percent was recommended when the negative definition of E-cadherin was determined according to the negative percentage of tumor cells in the immunohistochemical staining. However, large, well-designed prospective studies are required to further confirm our results.

## Supporting Information

Figure S1 **Egger's publication bias plot showed no publication bias for studies regarding the association of E-cadherin expression with overall survival (OS) in the meta-analysis: the relationship between the effect size of individual studies (HR, vertical axis) and the precision of the study estimate (standard error, horizontal axis).**
(TIF)

Figure S2 **Egger's publication bias plot showed no publication bias for studies regarding E-cadherin expression and differentiation grade in the meta-analysis.**
(TIF)

Figure S3 **Egger's publication bias plot showed the presence of publication bias for studies regarding E-cadherin expression and Dukes' stages in the meta-analysis.**
(TIF)

Figure S4 **Egger's publication bias plot showed no publication bias for studies regarding E-cadherin expression and lymphnode status in the meta-analysis.**
(TIF)

Figure S5 **Egger's publication bias plot showed no publication bias for studies regarding E-cadherin expression and metastasis in the meta-analysis.**
(TIF)

Table S1 **PRISMA checklist.**
(DOC)
