# Supplementary figures and images for: Downregulated E-Cadherin Expression Indicates Worse Prognosis in Asian Patients with Colorectal Cancer: Evidence from Meta-Analysis
Source: PLoS One. 2013 Jul 29;8(7):e70858. doi: 10.1371/journal.pone.0070858 (PMC3726621; doi:10.1371/journal.pone.0070858)

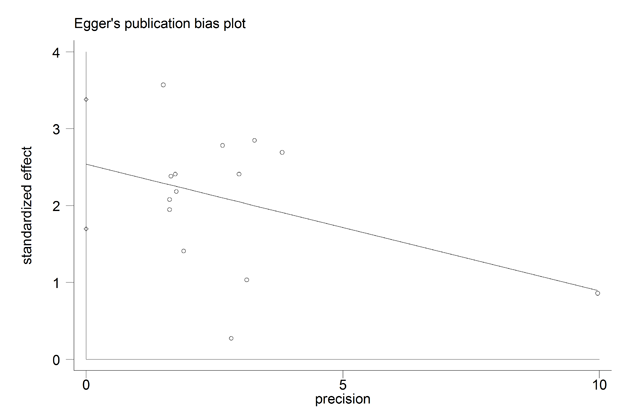

Supplement: Figure S1 — Egger's publication bias plot showed no publication bias for studies regarding the association of E-cadherin expression with overall survival (OS) in the meta-analysis: the relationship between the effect size of individual studies (HR, vertical axis) and the precision of the study estimate (standard error, horizontal axis). (TIF) [file pone.0070858.s001.tif]

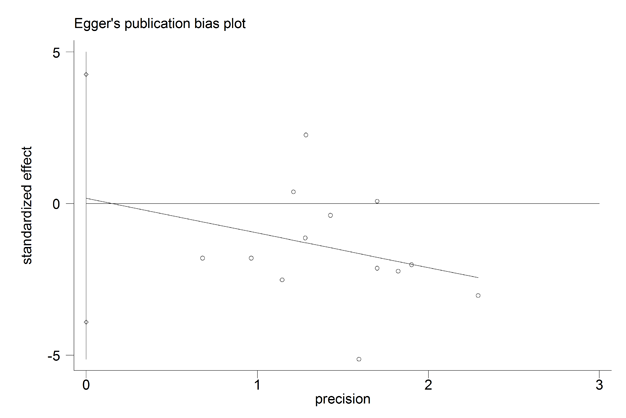

Supplement: Figure S2 — Egger's publication bias plot showed no publication bias for studies regarding E-cadherin expression and differentiation grade in the meta-analysis. (TIF) [file pone.0070858.s002.tif]

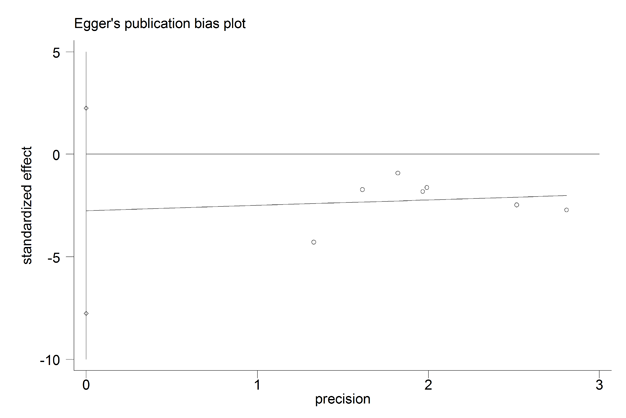

Supplement: Figure S3 — Egger's publication bias plot showed the presence of publication bias for studies regarding E-cadherin expression and Dukes' stages in the meta-analysis. (TIF) [file pone.0070858.s003.tif]

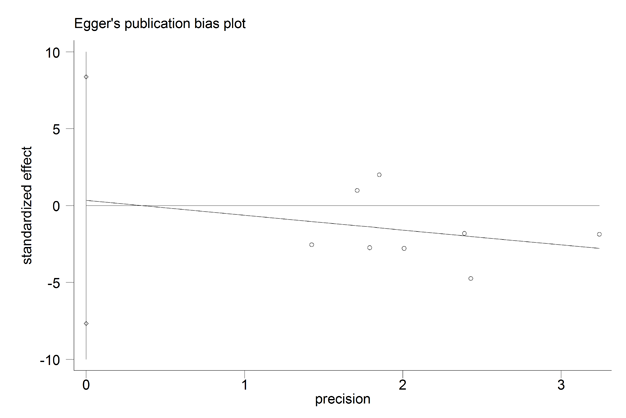

Supplement: Figure S4 — Egger's publication bias plot showed no publication bias for studies regarding E-cadherin expression and lymphnode status in the meta-analysis. (TIF) [file pone.0070858.s004.tif]

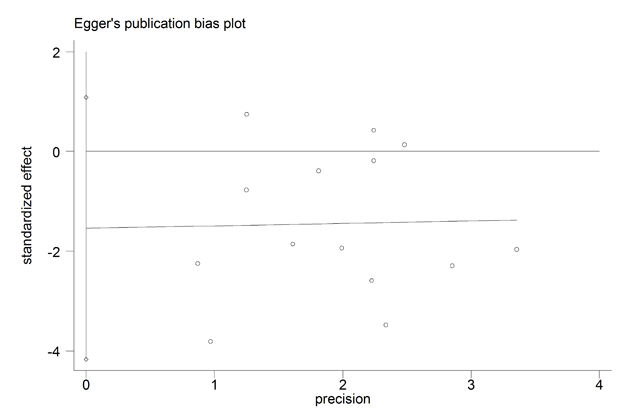

Supplement: Figure S5 — Egger's publication bias plot showed no publication bias for studies regarding E-cadherin expression and metastasis in the meta-analysis. (TIF) [file pone.0070858.s005.tif]
